# Surgical management of axillosubclavian vascular injuries

**DOI:** 10.12669/pjms.313.7316

**Published:** 2015

**Authors:** Muhammet Akyuz, Orhan Gokalp, Barcin Ozcem, Sedat Ozcan, Yuksel Besir, Ali Gurbuz

**Affiliations:** 1 Muhammet Akyuz, MD. Department of Cardiovascular Surgery, Katip Celebi University Ataturk Training and Research Hospital, Izmir, Turkey; 2 Orhan Gokalp, MD. Department of Cardiovascular Surgery, Katip Celebi University Ataturk Training and Research Hospital, Izmir, Turkey; 3 Barcin Ozcem, MD.N Department of Cardiovascular Surgery, Near East University, Lefkosa, Cyprus; 4 Sedat Ozcan, MD. Department of Cardiovascular Surgery, 18 Mart University Faculty of Medicine, Canakkale, Turkey; 5 Yuksel Besir, MD. Department of Cardiovascular Surgery, Katip Celebi University Ataturk Training and Research Hospital, Izmir, Turkey; 6 Ali Gurbuz, MD. Department of Cardiovascular Surgery, Katip Celebi University Ataturk Training and Research Hospital, Izmir, Turkey

**Keywords:** Injury, Vascular, Upper extremity, Surgery, Arterial trauma

## Abstract

**Objective:**

Complex surgical exposures to upper extremity injuries required for conventional surgery correlate with a high morbidity and mortality. We present our results with conventional surgery following injuries of the subclavian and axillary vessels.

**Methods:**

Between November 2007 and March 2012, 29 cases with subclavian-axillary vascular injury were operated. Diagnostic and treatment methods, associated organ injury, morbidity and mortality rates in these cases were respectively reviewed.

**Results:**

The causes of injuries were stab wounds in 11 cases (37.9%), gunshot wounds in 9 cases (31%), iatrogenic injuries in 5 cases (17.2%) and blunt trauma 4 cases (13.7%). Eight patients (27.5%) had isolated arterial injury while 21 patients (72.4%) had coexisting organ injury (vein, bone, soft tissue, nerve). Primary repair and usage of saphenous vein were the most common surgical methods. One patient died due to myocardial infarction. (Mortality 3.4%)

**Conclusions:**

Vascular injuries of axillosubclavian are frequently associated with neurogenic, osseous and soft tissue injuries and should have early intervention. Conventional surgery remains the choice of treatment in patients with poor status and urgency.

## INTRODUCTION

Peripheral vascular injuries are significant health problems in terms of mortality and limb loss. These injuries may cause limb loss most of the time; additionally, they may cause life-threatening, serious traumas when truncally located or out of control. The results of improper treatment of vascular traumas are limb loss, functional loss due to concomitant organ failure, graft thrombosis and pseudoaneurysm.^1^

Injuries of the subclavian artery are difficult to control and repair regarding complex anatomical structures and the coexistence of vital organs.^2^ These injuries increase mortality and morbidity, even causing sudden death.^2^,^3^ Early diagnosis with combination of physical examination and imaging techniques should be achieved and vascular repair should be carried out as soon as possible.

In this study, patients who underwent conventional surgery following injuries of the subclavian and axillary vessels were evaluated retrospectively.

## METHODS

Preoperative, perioperative and postoperative data of 29 cases who underwent surgery for injuries of subclavian-axillary vessels as a result of stab wounds, iatrogenic injury, shotgun wounds, and blunt trauma between November 2007 and March 2012 at the Izmir Katip Celebi University Atatürk Training and Research Hospital Cardiovascular Surgery Clinic were evaluated retrospectively.

The diagnoses were made with findings such as active hemorrhage, pulselessness, and ischemia of the limbs. Additional evaluation was made with direct graphy when there was suspected fractures with CT scan when there was suspected concomitant organ injury. Hemodynamically stabile patients with uncertain arterial injury underwent duplex ultrasonography and angiography prior to surgery.

Surgery was conducted with general anesthesia. Foley’s catheter was used for bleeding inhibition in 14 patients (48.2%). Compression with Foley catheter balloon is a safe and efficient method to stop bleeding.

During the operation, by clamping the proximal and distal ends of the vascular structures, hemorrhage was controlled following hemodynamic stabilization of the patient. Coexisting tendons, nerves and muscle injuries were consulted with concerned clinics perioperatively. Coexisting fractures or luxations were stabilized by reduction or by external/internal fixation after hemodynamic stabilization. Tube thoracotomy was applied when hemothorax-pneumothorax were considered.

All patients received heparin (80 IU/kg) injections before clamping vessels. Thrombectomy was performed, if necessary, prior to surgery. Venous injuries were either repaired by simple primary repair, end-to-end anastomosis or graft interposition, or ligatured. Fasciotomy was conducted when crush trauma was confronted. Low molecular weight-heparin or systemic heparin were given for 3-5 days postoperatively and warfarin was applied to patients with venous and polytetrafluoroethylene (PTFE) graft for 12 weeks. Prophylactic antibiotherapy and tetanus prophylaxis were applied.

## RESULTS

Twenty three patients (79.3%) were male and 6 (20.7%) were female, most of whom were between 15-30 years old (14 patients, 48.2%). The mean age was 34.71±14.83 (range, 15-74) years. Preoperative clinical and demographical data are shown in Table-I.

**Table-I:** Preoperative demographic data and clinical characteristics.

		n	%
Mean age (years)		34.71±14.83	
Gender (male)		23	79.3
Mechanism of injury			
	Stab wound	11	37.9
	Shotgun wound	9	31
	Iatrogenic	5	17.2
	Blunt trauma	4	13.7
Site of injury			
	Axillary artery	20	68.9
	Subclavian artery	9	31.1
Associated injury			
	Venous	15	51.7
	Bone	6	20.6
	Nerve	5	17.2

The most common symptoms were pain and motor-sensory loss. The most common findings were active bleeding, ischemia, pulselessness, and hypotension. The symptoms and findings are shown in Table-II.

**Table-II:** Symptoms and findings.

	n	%
Active bleeding	14	48.2
Hematoma	14	48.2
Pulselessness	18	62
Motor-sensory loss	13	44.8
Ischemia	19	65.5
Hypotension	15	51.7
Compartment syndrome	4	13.7
Hemopneumothorax	2	6.8

The subclavian artery was injured in 9 patients (31%) and the axillary artery was injured in 20 patients (69%). Eight patients (27.5%) had isolated arterial injury while 21 patients (72.4%) had coexisting organ injury (vein, bone, soft tissue, nerve.). In 16 patients (55.2%), arterial pathology was transection, and lacerations were detected in 13 patients (44.8%). Surgical techniques are listed in Table-III.

**Table-III:** Peroperative and postoperative clinical data.

		n	%
Type of incision			
	Transaxillary	13	44.8
	Combined(TA+IC)	3	10.3
	Infraclavicular	4	13.7
	Supraclavicular	3	10.3
	Combined (SC+IC)	2	6.8
	Thoracotomy	4	13.7
Surgical techniques (artery/vein)			
	Primary repair	10/7	34.4/24.1
	Saphenous vein interposition or repair	8/4	27.5/13.7
	PTFE graft interposition	9/1	31/3.4
	End to end anastomosis	2/2	6.8/6.8
	Ligation	0/1	0/3.4
ICU length of stay (days)		3.51 ± 2.72	
Hospital length of stay (days)		8.13±5.81	
Postoperative complications			
	Wound infection	3	10.3
	Graft thrombosis	2	6.8
	Partial neurological deficit	3	10.3
	Disarticulation	1	3.4
	Fasciotomy	5	17.2
	Mortality	1	3.4
Follow-up time(months)		42.17±18.9	

IC: infraclavicular, ICU: intensive care unit, SC: supraclavicular, TA: transaxillary.

Mean blood transfusion need was 5.93±4.39 (range, 1-20) IU. One patient (3.4%) died because of myocardial infection on the first postoperative day. Peroperative and postoperative clinical datas are listed in Table-III.

The mean follow-up period was 42.17±18.92 (range, 2-78) months. Seventeen patients attended periodic controls. In 17 patients (58.6%), no pathology was seen in duplex ultrasound (US). One patient (3.4%) had osteomyelitis and no vascular pathology. Seven patients (24.1%) were contacted by phone and it was confirmed that they had no symptoms.

## DISCUSSION

Vascular injuries, although seen only in 1-2% of cases, are remarkably important in terms of the potential risks of thrombosis, arteriovenous fistula, pseudoaneurysm, functional loss of extremity, amputation, and mortal complications such as infection and bleeding.^4^,^5^ Upper extremity injuries comprise 30% of total vascular injuries. Subclavian and axillary arteries are not commonly affected because of their localization. In addition the brachial artery is the most common artery subject to injury.^6^ In the literature, subclavian artery injuries can be classified with either brachiocephalic artery injuries or axillary artery injuries, thus different ratios may be detected.^7^,^8^ In our study, upper extremity injury rate was detected as 8.1% in the 6-year-old series.

Etiologic factors of upper extremity arterial injuries vary according to different studies.^9^,^10^ In the current study, the most common injury type was stab wounds with 11 patients (37.9%); 9 patients (31%) had shotgun wounds. Five patients (17.2%) had iatrogenic trauma and 4 patients (13.7%) were injured due to blunt trauma as a result of traffic accidents.

The diagnosis of peripheral arterial injuries is pronounced by physical examination.^11^ Furthermore, this diagnosis may be supported by invasive imaging techniques (for example, angiography) and non-invasive imaging techniques (for example, duplex ultrasonography.^12^,^13^ In the current cases, the diagnosis of arterial injury was made by physical examination, and supported by duplex ultrasound in 11 patients (37.9%) and by angiography in 3 patients (10.3%) who were hemodynamically stable. CT angiography showed abrupt cut-off at the axillary arteries.

In arterial injuries of the upper extremities, surgical techniques differ due to the lesions’ character. In vascular traumas, which are not appropriate for primary repair, autogenous grafts are preferred for vessel continuity.^14^,^15^ Autologous grafts are preferred for their short-term and long-term patency ratios (98%) and their resistance to infection.^16^ Synthetic grafts (PTFE and Dacron grafts) also have good results when autologous grafts are not available.^11^

In peripheral vascular traumas, both arteries and veins are injured most of the time.^4^ Nowadays, it is still debated whether to use ligation or reconstruction in venous injuries.^16^ The study of Bishara et al.^17^ regarding isolated venous injuries reveals that although transient extremity edema may be seen at a ratio of 35% in the early postoperative period, whether controlled by ligation or reconstruction, none of the cases had venous edema in the late term follow-up. Vascular trauma with bone fracture is reported in different studies. It is still debated as to whether vascular repair or orthopedic stabilization should be selected. It is a common choice that if the mobility of the bone is serious, the ischemic period is short, and hemodynamic stability is not disturbed, orthopedic stabilization should be handled first.^16^ Bleeding and wound infections are the most common complications reported in the literature.^16^ Mortality ratios were reported between 1.5% and 20% in different studies of vascular traumas.^11^,^18^ In the current study, one patient (3.4%) died due to myocardial infarction.

The emergence of endovascular modalities offers an alternative to traditional surgical management of select subclavian and axillary artery traumatic lesions.^19^ Endovascular repair of an injured axillosubclavian artery has been described in penetrating and iatrogenic injuries and, to a lesser extent, in cases of blunt trauma.^20^ As an effective therapy for traumatic arterial intima injury, arterial pseudoaneurysm, arteriovenous fistula, perforation and dissection. With the introduction of endovascular techniques for applications related to vascular injury, these less invasive modalities have increasingly been safely utilized in the treatment of select patients with a variety of peripheral vascular injuries.^21^ However, ideal patient selection requires additional investigation.

Complete vessel transection has been reported as a common cause for failure of an endovascular approach, primarily due to difficulty with crossing the complete transection and its associated hematoma.^22^,^23^ One of the disadvantages to the endovascular approach in treating axillosubclavian arterial transection from penetrating trauma is the concern for associated hematoma formation with resultant compression to the brachial plexus.^24^ As such, vessel transection has traditionally been approached with open vascular reconstruction.^19^

In summary subclavian and axillary arterial traumas cause high mortality and morbidity ratios because of concomitant organ damage while the correct diagnosis, rapid transportation, and appropriate intervention lowers mortality and morbidity ratios. Conventional surgery remains the choice of treatment in patients with poor status and urgency. However, hybrid prosedures in peripheral vascular surgery has increasingly become common. The patients admitted with emergency vascular injury become in more risk and more complicated. These patients often requires immediate intervention or at least don’t have time between the processes for different procedures. Morever, in patients proper and stable, hybrid procedures may provide successful treatment of associated vascular injury. We believe that conventional surgery procedures should continue to be first treatment option in most cases and that the hybrid procedures need to be limited with proper patient populations.
